# Low-Rate DoS Attacks Detection Based on MAF-ADM

**DOI:** 10.3390/s20010189

**Published:** 2019-12-29

**Authors:** Sijia Zhan, Dan Tang, Jianping Man, Rui Dai, Xiyin Wang

**Affiliations:** College of Computer Science and Electronic Engineering, Hunan University, Changsha 410082, China

**Keywords:** low-rate denial of service attacks, anomaly detection, adaptive fusion of multiple features, time-frequency joint distribution, isolation trees

## Abstract

Low-rate denial of service (LDoS) attacks reduce the quality of network service by sending periodical packet bursts to the bottleneck routers. It is difficult to detect by counter-DoS mechanisms due to its stealthy and low average attack traffic behavior. In this paper, we propose an anomaly detection method based on adaptive fusion of multiple features (MAF-ADM) for LDoS attacks. This study is based on the fact that the time-frequency joint distribution of the legitimate transmission control protocol (TCP) traffic would be changed under LDoS attacks. Several statistical metrics of the time-frequency joint distribution are chosen to generate isolation trees, which can simultaneously reflect the anomalies in time domain and frequency domain. Then we calculate anomaly score by fusing the results of all isolation trees according to their ability to isolate samples containing LDoS attacks. Finally, the anomaly score is smoothed by weighted moving average algorithm to avoid errors caused by noise in the network. Experimental results of Network Simulator 2 (NS2), testbed, and public datasets (WIDE2018 and LBNL) demonstrate that this method does detect LDoS attacks effectively with lower false negative rate.

## 1. Introduction

Denial of service (DoS) attacks have always been the main threats to network security [1]. In February 2019, the website of the Philippine National Association of Journalists suffered a DoS attack and was closed for 12 h. The Facebook was also attacked by DoS in March 2019, and users could not log in to their accounts. Nowadays cloud computing [2], software defined network [3,4], and wireless sensor networks [5,6] are widely applied. The development of these technologies makes the current network structure which has higher node density, larger scale and limited resources more vulnerable to DoS attacks [7,8,9]. This situation is even worse when more and more variants of DoS attacks arise [10,11]. Low-rate denial of service (LDoS) is a smart attack unlike the flooding attacks due to its stealthy and low-rate attack traffic behavior. It sends periodical packet bursts to attack legitimate flows by exploiting the vulnerability of transmission control protocol (TCP) adaptive mechanism [12]. Therefore, it is fairly simple for LDoS attacker to elude the existing counter-DoS mechanisms [13].

Existing researches [14] indicate that the network traffic is actually a non-stationary signal due to the unpredictable change of the network at all times. The anomalies of network traffic caused by LDoS attack flows may indicate in the time domain, such as the traffic reduced by fake congestion. They may also be expressed in the frequency domain, such as periodicity, abnormal frequency distribution of the traffic, and so on. However, these existing LDoS detection algorithms are only based on the characteristics in the time domain [15,16,17,18] or the frequency domain [2,19,20]. Another limitation associated with the emerging literature is that not enough attention has been paid to the adaptive ability of detection schemes to scenes and the filtering of noise in the environment.

In response to the above limitations, we propose an anomaly detection method based on adaptive fusion of multiple features (MAF-ADM) for LDoS attacks. Time-frequency joint analysis, a powerful tool for analyzing non-stationary signals, is used to analyze the anomalies of network traffic caused by LDoS attack streams. Several statistical metrics of the time-frequency joint distribution are chosen to generate isolation trees. Then anomaly score is calculated as the basis of LDoS attack detection.

The major contributions of our work are threefold. Firstly, we analyze network traffic in time-frequency domain and introduce a series of novel features for detecting LDoS attacks. These attributes can simultaneously reflect the anomalies in time domain and frequency domain. By evaluating on Network Simulator 2 (NS2) simulations, these attributes do have good sensibility to identify LDoS attacks of different parameters. Secondly, we establish isolation trees for the detection metrics and then fuse them together to describe the network state by linear weighted way. The weight of each isolation tree is dynamically adjusted according to their ability to isolate the LDoS attacks. By this way, the adaptability and accuracy of the method is further improved. Thirdly, we apply the weighted moving average algorithm to filter noise so that the method has lower false positive rate.

The rest of the paper is organized as follows: Section 2 introduces related researches about characteristics and detection methods of LDoS attacks in recent years. Section 3 describes the detection metrics based on time-frequency analysis. A new detection algorithm based on MAF-ADM is proposed in Section 4. The performance of MAF-ADM is tested on simulation experiment NS2, testbed, and the public datasets in Section 5. In Section 6, we summarize this paper and introduce the future work.

## 2. Related Researches

### 2.1. Characteristics of LDoS Attacks

**Figure 1:**
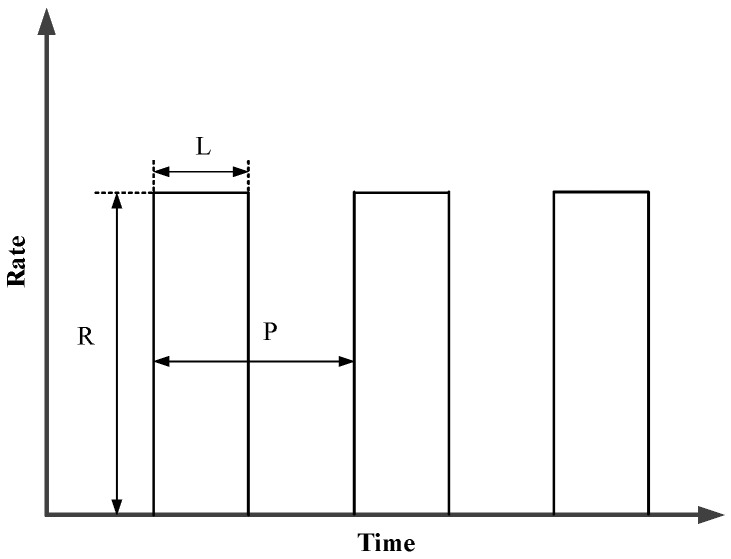
Model of low-rate denial of service (LDoS) attacks.

LDoS attack flow has a lower average rate than the traditional DoS attack flow, which makes it more insidious and difficult to be detected [21]. LDoS attacks send periodical packet bursts with model as shown in Figure 1 [22]. *P* represents the attack period, *R* is the attack rate, and *L* denotes the duration of a single attack pulse.

It repeatedly evokes adaptive adjustment of TCP congestion control so that the network is in a fake congestion state when the attack strength (R∗L) is large enough. Depending on the adaptive mechanism evoked by the attack, LDoS attacks can be divided into retransmission timeout (RTO)-based attacks and additive increase and multiplicative decrease (AIMD)-based attacks.

**Figure 2:**
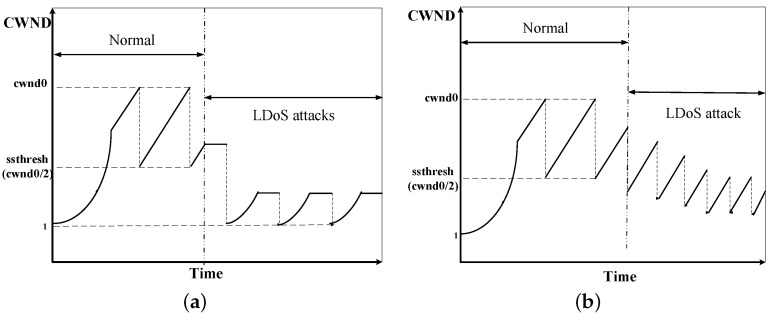
Transmission control protocol (TCP) link state of LDoS attacks based on different congestion control mechanisms: (**a**) shows congestion window (CWND) under retransmission timeout (RTO)-based LDoS attacks, (**b**) depicts CWND under additive increase multiplicative decrease (AIMD)-based LDoS attacks.

- RTO-based LDoS attacks: A TCP sender normally sets retransmission timeout (RTO) for each packet. As shown in Figure 2a [23], when the network link is in normal state, we can assume that RTO of the sender is the minimum value (usually set to 1 s in order to achieve optimal throughput of the network). But when an attack pulse is arrived, the TCP gets into the timeout retransmission state. During the attack interval, the sender begins to get into the slow start and successfully retransmits. For some data packets, the RTO can also return to the minimum value by Formula (1) [24]. *G* is the clock granularity. SRTT and VRTT represent round-trip time and the variation of round-trip time respectively. The above process repeats so that the quality of network services is reduced.
  (1) $$\begin{array}{cc} RTO & =min\left\{RTO_{max},max\left\{RTO_{min},\right.\right.\\ & \;\left.\left.SRTT+max\left(G,4\times VRTT\right)\right\}\right\} \end{array}$$
- AIMD-based LDoS attacks: The additive increase and multiplicative decrease (AIMD) mechanism is to resend the packet immediately after the sender receives three duplicate acknowledge characters (ACKs), which reduces its congestion window (CWND) through multiplicative decrease (MD) algorithm and increases the CWND according to additive increase (AI) algorithm. The link is always in the AIMD state and does not enter the timeout retransmission and slow start under the AIMD-based LDoS attacks as Figure 2b [25] presented. But its CWND is decreasing so that the system performance is gradually reduced. Finally the CWND is reduced to a limit and the system performance is the worst, which cannot be recovered by itself.

**Figure 3:**
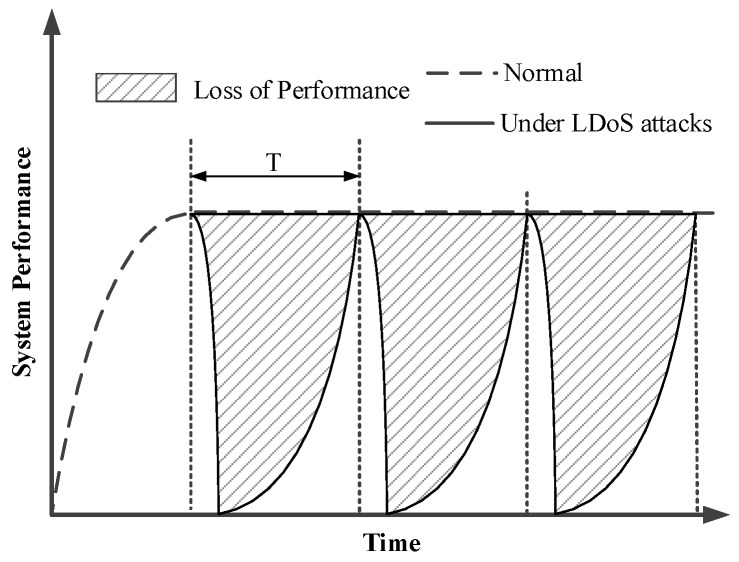
The impact of LDoS attacks on system performance.

Both of the above LDoS attacks exploit the TCP adaptive mechanism. The LDoS attacker usually chooses user datagram protocol (UDP) stream to launch the attack. Even if the network sends a congestion indication (such as packet loss, repeated ACKs etc.), UDP does not reduce the number of packets sent to the network but TCP does. Under LDoS attacks, the attack pulse stream preempts more and more network resources, and the victim believes that the network is “congested” and rapidly reduces its transmission rate. The quality of service in the network is seriously reduced as Figure 3 [26] showed. Therefore, how to detect LDoS attacks is a very important issue for network security.

### 2.2. Detection of LDoS Attacks

There are various LDoS detection algorithms proposed in recent years. Most of them can be roughly classified into time domain and frequency domain according to detection characteristics.

- Time domain based detection algorithm
  Meng et al. [27] established a feedback control model to describe the process of random early detection(RED) congestion control, by which the congestion window and router queue behaviours were analyzed together. Then a queue distribution model consisted of the instantaneous queue and the average queue was proposed to extract the attack feature. After that, a simple distance-based approach and an adaptive threshold algorithm were combined to detect every LDoS attack burst. The experimental results of NS2 and testbed proved that LDoS attack bursts can almost be detected completely and this method was especially robust to legitimate short bursts.
  Wu et al. [28] proposed a detection algorithm based on the multifractal characteristics of network traffic. It was proved that the multifractal characteristics of network traffic are different between the states of normal and LDoS attacks by using MF-DFA algorithm. Then the wavelet point-by-point estimation algorithm was used to calculate the $H\ddot{o}lder$ exponent to determine when the attack begins and ends. The NS2 results showed that the approach could achieve the detection probability of 92% and false positive rate of 9%.
  Guo et al. [29] presented a situation aware method based on multi-feature adaptive fusion to detect LDoS attacks in the border gateway protocol (BGP) routing system. The statistical characteristics of BGP routing information such as frequency of announce messages, frequency of withdraw messages and average autonomous systems (AS) path length were selected as representative of security state of the system. Each of the above features was modeled by reverse cloud generation algorithm, and then the dynamic weights were used to fuse the submodel. Experiment results showed that this method can effectively detect not only control plane attacks and but also data plane attacks (BGP-LDoS).
  Tang et al. [30] applied the two steps cluster to analyze network traffic on a large time scale. According to the characteristics of TCP traffic was abnormal when the LDoS attack occured, the abnormal cluster was further detected by using the concept of data slice from a small time. Experimental results on NS2 and public datasets Lawrence Berkeley National Laboratory (LBNL) and Measurement and Analysis on the WIDE Internet (WIDE2018) showed that LDoS attacks could be effectively detected.
- Frequency domain based detection algorithm
  Neha et al. [2] proposed an algorithm for detecting and filtering LDoS attack streams in the frequency domain. This method based on power spectral density was used to monitor the aggregated flow in the cloud network in real time. The method could significantly reduce the possibility of attack in a real cloud environment based on OpenStack.
  Chen et al. [23] combined power spectral density to propose two new information features for detecting LDoS attacks, which named Fourier power spectrum entropy and wavelet power spectrum entropy. Based on these two information features, a Robust-RED queue management algorithm based on power spectral density was proposed to filter the LDoS attack streams. The algorithm was verified on the NS3 simulation experiment platform, which could indeed resist different LDoS attacks.
  Wu et al. [20] also proposed a method based on frequency spectral analysis for detecting and filtering LDoS attack streams. The TCP streams and LDoS attack streams were transformed from time domain to frequency domain and obtained the round-trip time according to the frequency domain search algorithm. It was found that the magnitude of energy of TCP stream is mainly concentrated in the points of n/RTT. According to this feature, an infinite impulse response filtering algorithm was proposed, which can filter LDoS attack flows with as little impact as possible on legitimate TCP flows.
  Wu et al. [31] applied Kalman filter to detect LDoS attacks. By analyzing the characteristics of victim network traffic at the beginning of LDoS attacks, the error between one step prediction and the optimal estimation was used as the basis for detection.

These existing detection methods still have some deficiencies, such as (1) high false negative rate caused by using only the characteristics of time domain or frequency domain; and (2) lack of processing of network traffic noise and adaptability. For example, the key parameters such as the detection threshold depend on experience and cannot be adjusted according to the change of network environment.

To address the above limitations, a new algorithm for detecting LDoS attacks is proposed in this paper. This study is based on the fact that the time-frequency joint distribution characteristics of legitimate TCP traffic will be changed by the LDoS attacks flow. The detection features are more robust to detect different LDoS attacks since the time-frequency joint distribution can simultaneously reflect the anomalies in time domain and frequency domain. Then the anomaly score is calculated by MAF-ADM to metric that change, which is the basis of detecting LDoS attacks.

## 3. Time-Frequency Joint Analysis Based Detection Metrics

In this section, we firstly describe that how to obtain time-frequency joint distribution by performing short-time Fourier transform on network traffic in the bottleneck link. The reason for that is the network will be in a state of fake congestion and network traffic in the bottleneck link will be the first to bear the brunt when an LDoS attack occurs. Some statistical features of the time-frequency joint distribution are extracted as detection features, which accurately represent the anomalies caused by LDoS attacks both in frequency domain and in time domain [32].

### 3.1. STFT Analysis of Network Traffic

In this paper, detection window is used as the basic unit for detecting LDoS attacks. Detection window is defined as a sample sequence consisting of network traffic samples $x\left(\tau \right)$ that are continuously acquired over a given length of time.

Given window function of fixed time width $w\left(t\right)$ that slides along the time axis $x\left(\tau \right)$, the short-time Fourier transform (STFT) of the signal is defined as Formula (2) [33].

(2) $$STFT_{x}\left(t,f\right)=\int_{-\infty }^{\infty }x\left(\tau \right)w^{{}^{\prime }}\left(t-\tau \right)e^{-2j\pi f\cdot \tau }d\tau$$

Considering that the time series $x\left(\tau \right)$ of sampling network traffic is in discrete form, it is necessary to discrete transformation. We set *t* and *f* as the sampling intervals of time variable and frequency variable respectively, and *N* is the total number of samples of the time series $x\left(\tau \right)$, m,n=1,2,…,N. The discrete form of the sequence’s STFT is defined as Formula (3) [33].

(3) $$STFT_{x}\left(m,n\right)=\sum_{k=1}^{N}x\left(k\right)w^{{}^{\prime }}\left(k-m\right)e^{-2j\pi nk/N}$$

The result $STFT_{x}\left(m,n\right)$ of the transformation obtained by the equation is a two-dimensional complex matrix. The rows *m* and columns *n* of the matrix correspond to the sampling point of time and frequency respectively. The elements in the matrix correspond to the spectral amplitude. The magnitude matrix can be expressed as Formula (4).

(4) $$A\left(m,n\right)=\left|STFT_{x}\left(m,n\right)\right|$$

### 3.2. Time-Frequency Joint Distribution Based Detection Metrics

The matrix $A\left(m,n\right)$ is essentially the energy distribution of the signal at different frequencies of different times. In this subsection, by using NS2, we built a dumbbell network topology as the same as Section 5.1.1 and selected two kinds samples (normal samples and samples containing LDoS attacks) for analyzing the anomalous characteristics of the time-frequency joint distribution of TCP traffic caused by LDoS attack flows.

#### 3.2.1. Total Signal Energy

**Figure 4:**
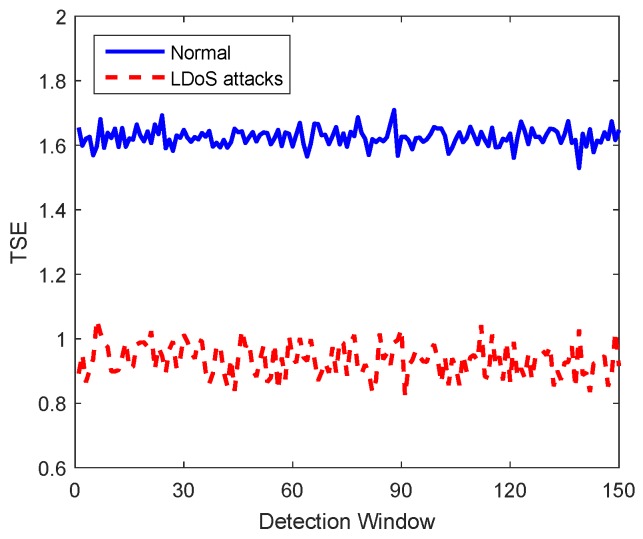
Total signal energy comparison.

The total signal energy (TSE), named *T*, refers to the sum of the amplitude frequency of all elements in the time-frequency joint distribution matrix as Formula (5). In Figure 4, the total signal energy values of 150 detection windows acquired in normal state and LDoS attack state respectively are compared. Due to the constant preemption of resources by the LDoS attack stream, the service quality of TCP connection in the network is affected. Therefore, the average value of TCP is lower, and the value of TSE is also reduced according to Formulas (4) and (5).

(5) $$T=\sum_{i=1}^{N}\sum_{j=1}^{\frac{1}{2}N}A\left(i,j\right)$$

#### 3.2.2. Segmentation Frequency Ratio

The segmentation frequency ratio (SFR), expressed as $S=\langle S_{Low},S_{MidLow},S_{MidHigh},S_{High}\rangle$, mainly reflects the frequency distribution of the original signal. We divide the time-frequency joint distribution matrix from the highest frequency to the DC part into four parts according to the ratio of 1/2, 1/4, 1/8, 1/8, which including high frequency, medium high frequency, medium low frequency, and low frequency. This division is based on the fact that the anomalies in the low frequency part are more obvious and require further subdivision. Thus we take $S_{Low}$ as an example to illustrate the calculate process as Formula (6).

(6) $$S_{Low}=\frac{1}{T}\sum_{i=1}^{N}\sum_{j=1}^{\frac{1}{16}N}A\left(i,j\right)$$

**Figure 5:**
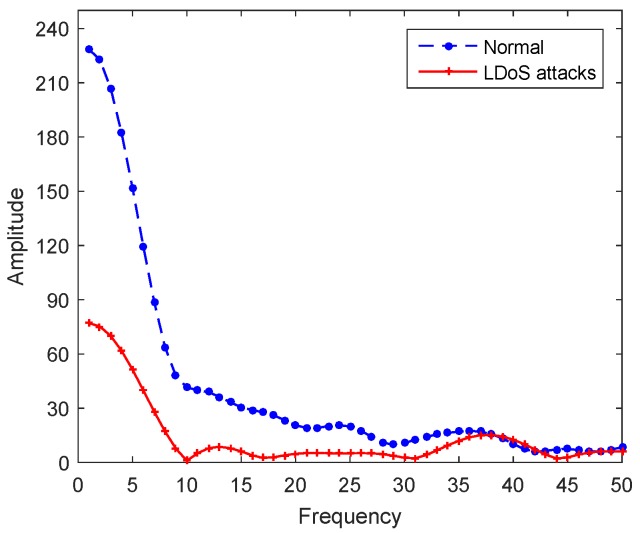
Instantaneous frequency comparison.

Figure 5 shows the instantaneous frequency comparison at between a certain moment under normal state and LDoS attack state. The network traffic is stable and the fluctuation is small in normal state, which concentrated in the low frequency part. But the pulse attack flow makes the network links consecutively switching between states of overload and underload. The congestion control mechanism is triggered repeatedly so that the TCP traffic is in “up” and “down” repeatedly and dramatically. Therefore, the SFR is more even in LDoS attack state.

#### 3.2.3. Normalized Variance of Segmentation Frequency

The normalized variance of segmentation frequency (NVSF), denoted by $N=\langle N_{Low},N_{MidLow},N_{MidHigh},N_{High}\rangle$, mainly reflects the fluctuation of energy in the frequency part. The normalized variance is the variance obtained by dividing each element by the mean of all elements of the entire time-frequency joint distribution matrix. For the same reason that the signal in the low frequency part is more concentrated so that the change is more obvious, the division of the frequency part is consistent with the division in SFR. Then how to calculate $N_{Low}$ is shown as the following Formula (7).

(7) $$\begin{array}{cc} N_{Low} & =\frac{N^{2}}{2T}\cdot \sqrt{\frac{16}{N^{2}}\cdot \sum_{i=1}^{N}\sum_{j=1}^{\frac{1}{16}N}\left(A\left(i,j\right)-\frac{2T}{N^{2}}\right)}\\ & =\frac{2N}{T}\cdot \sqrt{\sum_{i=1}^{N}\sum_{j=1}^{\frac{1}{16}N}\left(A\left(i,j\right)-\frac{2T}{N^{2}}\right)} \end{array}$$

**Figure 6:**
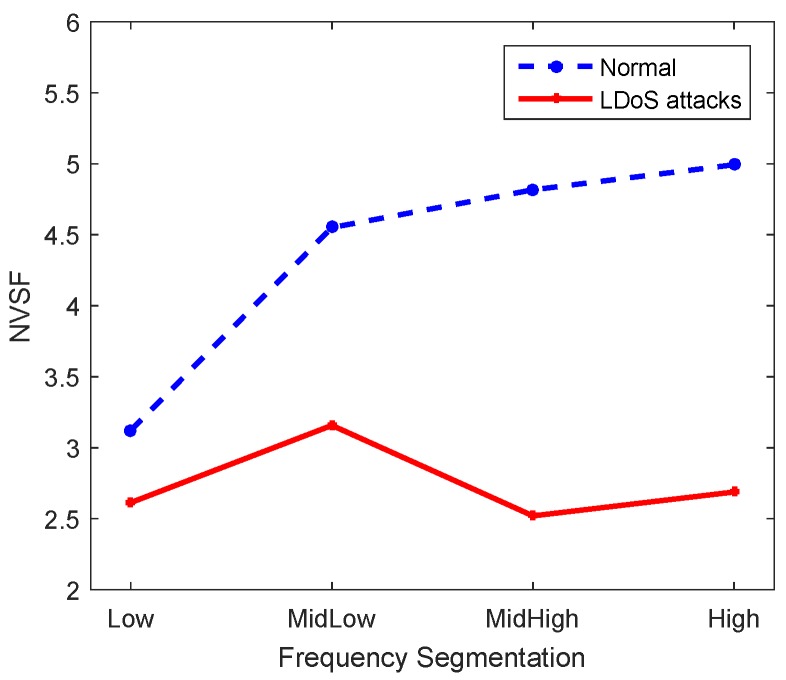
Normalized variance of segmentation frequency comparison.

Normalized variance comparison of each frequency part between detection window in normal state and LDoS attack state as shown in Figure 6. The segmentation frequency distribution in the normal state is more concentrated, while the distribution of each frequency part is more even in LDoS attack state.

## 4. LDoS Attacks Detection Method

**Figure 7:**
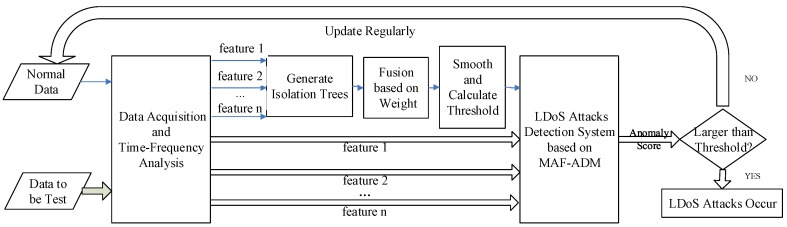
The processing flow of LDoS detection method based on MAF-ADM.

In this section, we present MAF-ADM for detecting LDoS attacks as shown in Figure 7, which achieves transition between features of network traffic and anomaly score of network state. This study is based on isolation forest which is an excellent anomaly detection method purely based on concept of isolation without employing any distance or density measure. We firstly utilize the features of time-frequency joint distribution to generate isolation trees for normal state (traffic data under normal state that has been collected from bottleneck links in the network), and then fuse all isolation trees into an isolation forest through linear weighted manner. With the isolation forest, we can evaluate the anomaly score by weighted moving average algorithm to judge whether LDoS attacks occur.

### 4.1. Generate Isolation Trees

As analyzed above, we can utilize the features of time-frequency joint distribution to describe the possibility of the network suffering from LDoS attacks. It is costly that simply combined these features to construct a multi-dimension description model. Therefore, we build isolation trees for these features, which have a low linear time complexity and a small memory requirement.

Supposing $Y=\left\{y_{i}\right\},y_{i}=\langle T,S,N\rangle ,i=1,2,\ldots ,n$ is the detection metrics of training data with *d* characteristic dimension, the binary tree structure named isolation tree is used to separate samples containing LDoS attacks from normal samples.Since samples that containing LDoS attacks usually have the characteristics of being sparsely distributed and distant from dense normal samples, they are closer to the root node in the isolation tree structure and therefore more easily isolated.

The construction steps [34] of isolation trees are that randomly selecting feature *q* and its value *p* to recursively split the training data *Y* until one of the following three conditions is met:

- The isolation tree reaches a defined height;
- There is only one sample on the node;
- Features of all the nodes are the same.

### 4.2. Linear Weighted Fusion

In [34], path length $h_{j}\left(y_{i}\right)$ is defined as the number of edges traversed by the sample $y_{i}\epsilon Y$ from the root node to the external node in the $j_{st}$ isolation tree, which describes its deviation from normal state. However, the strategy of randomly selecting features and dividing feature values may make some isolation trees not equipped with the ability to distinguish between normal samples and samples containing LDoS attacks.

**Figure 8:**
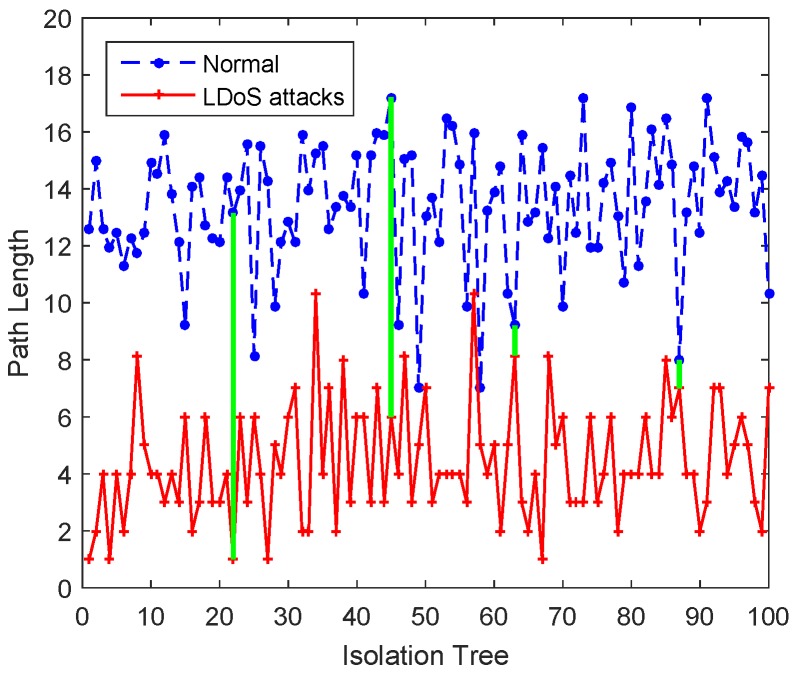
Path length of two kinds samples in the isolation trees.

For the purpose of analyzing the ability of isolation trees to isolate samples containing LDoS attacks, we also used two kinds of samples (normal samples and samples containing LDoS attacks) to calculate the path length in each isolation tree. Figure 8 proves that the ability of each isolation tree to isolate abnormal samples is not the same. For example, in the $22_{st}$ isolation tree and the $45_{st}$ isolation tree, two samples is widely separated, while in the $63_{st}$ isolation tree and the $87_{st}$ isolation tree, the two samples are too close to be indistinguishable.

Path length in different tree structures is not comparable, so anomaly score *S* is proposed to fuse the normalized results of all isolation trees. It ignores the difference between isolation ability of isolation trees that using mean value of the path length to calculate anomaly score. In order to more rationally synthesize the result of each isolation tree, we apply the weighted path length to instead of the mean value. Then the weight of the $j_{st}$ isolation tree is obtained by Formula (8).

(8) $$w_{j}^{cur}=\lambda d_{j}+\left(1-\lambda \right)w_{j}^{pre}$$

where $w_{j}^{cur}$ is the current weight of the $j_{st}$ isolation tree. $w_{j}^{pre}$ is the previous weight. $\lambda \in \left[0,1\right]$ is used to control the speed of weight updating so that this method can be adaptive to scene change. $d_{j}$ is the isolation ability of the $j_{st}$ isolation tree at present, which can be calculated as Formula (9). There are a total of *t* isolation trees.

(9) $$d_{j}=\frac{h_{j}}{\sum_{m=1}^{t}h_{m}}$$

Then the anomaly score can be calculated by Formula (10).

(10) $$S\left(y_{i},n\right)=2^{-\frac{1}{c\left(n\right)}\cdot \sum_{k=1}^{t}w_{k}^{cur}\cdot h_{k}\left(y_{i}\right)}$$

where $c\left(n\right)$ is the average depth of isolation trees.It is used to normalize the result and its calculation Formula (11) [35] is as follows.

(11) $$c\left(n\right)=2H\left(n-1\right)-\frac{2}{n}\left(n-1\right)$$

$H\left(i\right)$ is the harmonic number and can be estimated by Euler’s constant as Formula (12).

(12) $$H\left(i\right)=ln\left(i\right)+0.5772156649$$

### 4.3. Discrimination of LDoS Attacks

**Figure 9:**
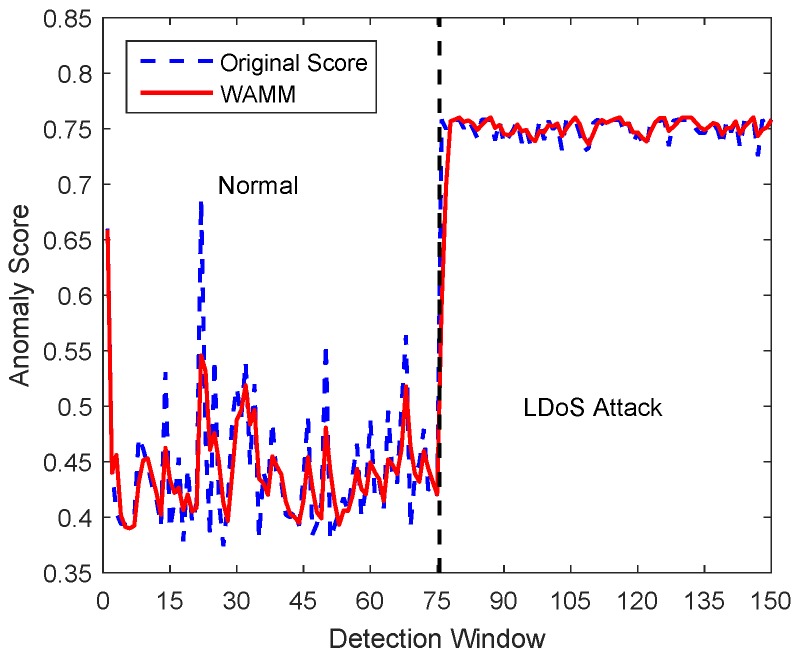
Smoothed anomaly score compared to the original value.

Network traffic has randomness, which means that many accidental factors, such as data stream bursts, data stream silence and occasional noise, may easily cause false positives. To solve this problem, we adopt weighted moving average algorithm to smooth anomaly score as Formula (13). Anomaly score before the current detection window is used to represent the abnormality degree of the current detection window. As Figure 9 shown, the curve of anomaly score smoothed by the weighted moving average algorithm is smoother, so that the false alarm can be effectively reduced.

(13) $$\bar{S}\left(y_{i},n\right)=\sum_{k=t-N+1}^{t}\alpha_{k}S\left(y_{k},n\right)$$

$\alpha_{k}$ is the weight of detection window *k* as Formula (14). Considering that the values of adjacent windows are similar, the larger weight is given to the nearer detection window so that the smoothed value is closer to the real value.

(14) $$\alpha_{k}=\left(k-t+N\right)/\sum_{i=1}^{N}i$$

Then the criterion for determining whether the sample $y_{i}$ includes LDoS attacks is as follows:

- When $\sum_{k=1}^{t}w_{k}^{cur}h_{k}\left(y_{i}\right)\to c\left(n\right)$, $\bar{S}\left(y_{i},n\right)\to 0.5$, that means all samples in the data set do not contain obvious LDoS attacks;
- When $\sum_{k=1}^{t}w_{k}^{cur}h_{k}\left(y_{i}\right)\to 0$, $\bar{S}\left(y_{i},n\right)\to 1$, that means the sample includes LDoS attacks;
- When $\sum_{k=1}^{t}w_{k}^{cur}h_{k}\left(y_{i}\right)\to n-1$, $\bar{S}\left(y_{i},n\right)\to 0$, that means the sample is normal.

The anomaly score calculated based on the above algorithm is a continuous value between 0 and 1, so we need a threshold to divide whether the LDoS attack occurs. The anomaly scores will be approximately normal distribution when the number of samples is sufficient according to the Central Limit Theorem.

Therefore, the threshold can be calculated as Formula (15). The given constant *z* in the confidence interval is related to detection accuracy, which is set to 2.58 in this paper. Then the sample $y_{i}$ will be judged as LDoS attacks when its anomaly score is large than the threshold.

(15) $$Threshold=Mean\left(\bar{s}\right)+z\cdot Std\left(\bar{s}\right)$$

## 5. Experiments and Results Analysis

In this section, we verified the detection performance of this method on NS2 [36], testbed, and public datasets [37,38]. Experiments of NS2 and testbed were used to verify the stability and accuracy of the method for detecting LDoS attacks. Experiments on the public datasets were used to evaluate the false positive rate of the method in complex network environment. Indexes used to evaluate the detection performance are detection accuracy, false negative rate and false positive rate.

### 5.1. Experiments on NS2

#### 5.1.1. The Experimental Environment

**Figure 10:**
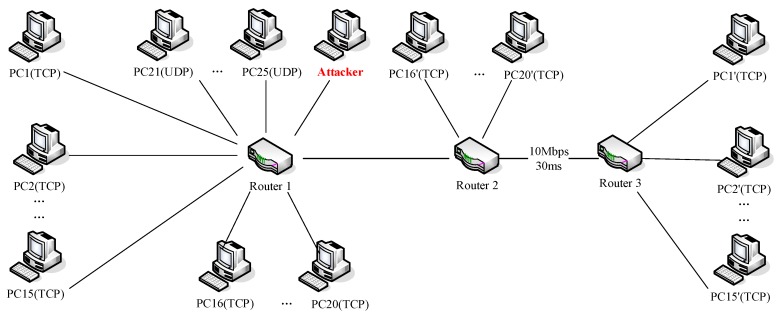
Topology of Network Simulator 2 (NS2) simulation experiment platform.

We built a dumbbell-type network topology by NS2 as shown in Figure 10. There were a total of 25 legal flows in the network, which included 15 TCP flows, five TCP flows and tive UDP flows for generating background traffic. Router two and Router three were connected by a bottleneck link with a bandwidth of 10 Mbps and a delay of 30 ms. Except for that bottleneck link, all other links had a bandwidth of 100 Mbps and a delay of 15 ms. All TCP flows used the New Reno congestion control protocol with RTO set to 1.0 s. All routers used RED as the queue management algorithm. Other parameters were the default parameters of NS2 platform.

In this topology, legitimate users communicated with others by using TCP connections and UDP connections. LDoS attacker usually used UDP protocol to send periodic pulse streams. In Router three, we extracted and sampled the packet arrival number of TCP at a period of 0.1 s to obtain the time series data. The duration of detection window was set to 10 s.

#### 5.1.2. Performance of LDoS Attacks Detection

**Figure 11:**
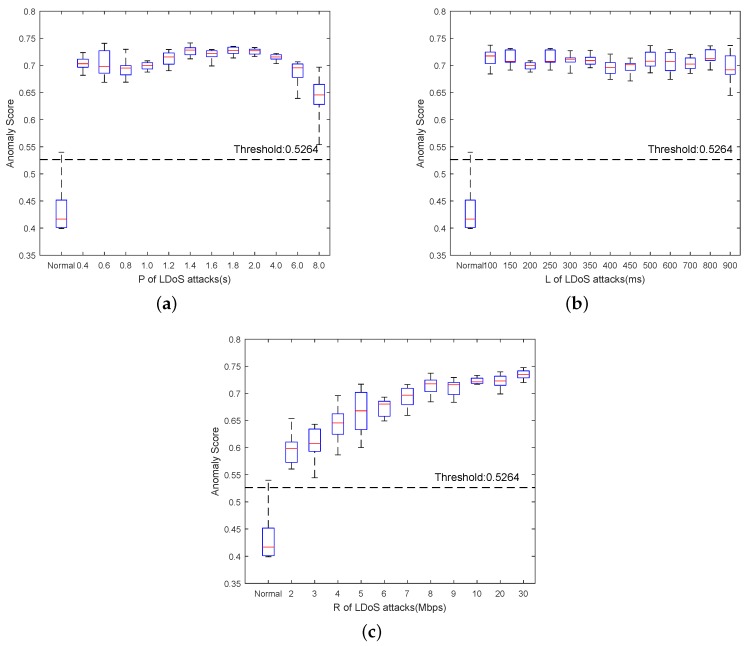
Anomaly score between normal state and LDoS attacks of different parameters: (**a**) shows LDoS attack of R = 8 Mbps, P = [0.4,8] s, L = 0.2 s compare with the normal state, (**b**) depicts LDoS attacks of R = 8 Mbps, P = 1 s, L = [0.1,0.9] s compare with the normal state, and (**c**) presents LDoS attacks of R = [2,30] Mbps, P = 1 s, L = 0.1 s compare with the normal state.The boxes include maximum value, 75th percentile, median value, 25th percentile, and minimum value of anomaly score under different LDoS attacks. The red lines in all the boxes are the median values.

Based on the analysis in Section 2.1, we conducted multiple groups of simulations to evaluate our method for detecting LDoS attacks of different parameters. The specific settings of the attack parameters are shown in Table 1. The anomaly score of normal network traffic was applied to determine an appropriate threshold for detecting the LDoS attacks. The state of detection window was identified as LDoS attacks when its anomaly score was larger than 0.5264. From G2 to G4, we set controlled experiments of the LDoS parameters respectively. The variation range of anomaly scores under different attack parameters was calculated as Figure 11.

Figure 11a,b present that the anomaly score distribution of network traffic under LDoS attacks did not vary a lot when P and T changed. From Figure 11c, we can observe that the anomaly score distribution of the normal network traffic was closest to that of network traffic under LDoS attacks with R = 2 Mbps. The reason for that is the low ratio of attack rate of LDoS attack stream to the bottleneck link bandwidth. When the LDoS attack stream only has a weak advantage to compete with the legitimate TCP stream for resources, it is difficult to cause the link congestion to reduce the quality of service.

**Figure 12:**
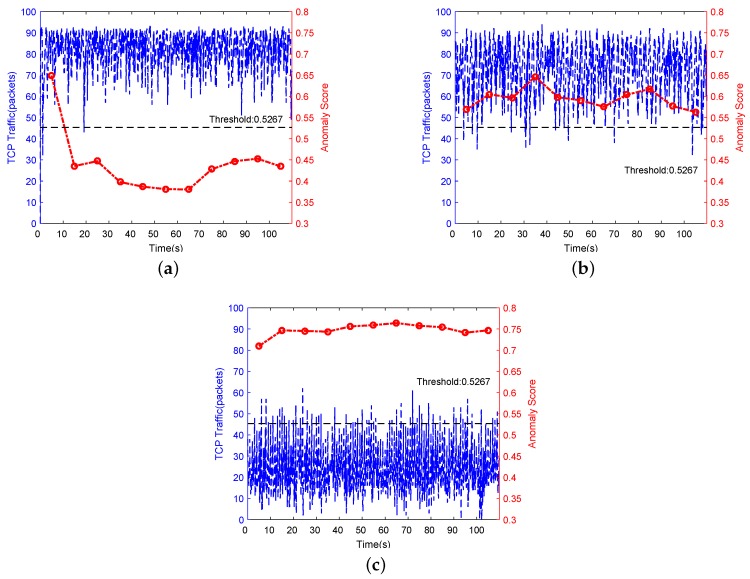
Distributions of TCP traffic and anomaly score between normal and LDoS attacks: (**a**) is under the state of normal, (**b**,**c**) are under LDoS attacks with R = 2 Mbps, P = 1 s, L = 0.1 s and R = 30 Mbps, P = 1 s, L = 0.1 s respectively.

We further compared TCP traffic and abnormal scores of the normal state, LDoS attacks with (R = 2 Mbps, L = 0.1 s, P = 1 s) and LDoS attacks with (R = 30 Mbps, L = 0.1 s, P = 1 s), as shown in Figure 12. These results seem consistent with our study. For example, Figure 12a,b shows the distribution of TCP traffic and anomaly score between the normal state and LDoS attack with (R = 2 Mbps, L = 0.1 s, P = 1 s) is very similar, which means the LDoS attack effect was very weak so that it almost could not reduce the quality of network service. In addition, the 1st detection window of the normal state was misjudged as under LDoS attack, the reason is that the network at this time was in a state of just establishing TCP connections, and since the traffic distribution was similar to the state under LDoS attack, false alarm occurred. Figure 12c was under the strongest attack, the quality of service was severely reduced, and therefore anomaly score was the highest.

### 5.2. Experiments on Testbed

#### 5.2.1. Testbed Experimental Environment

**Figure 13:**
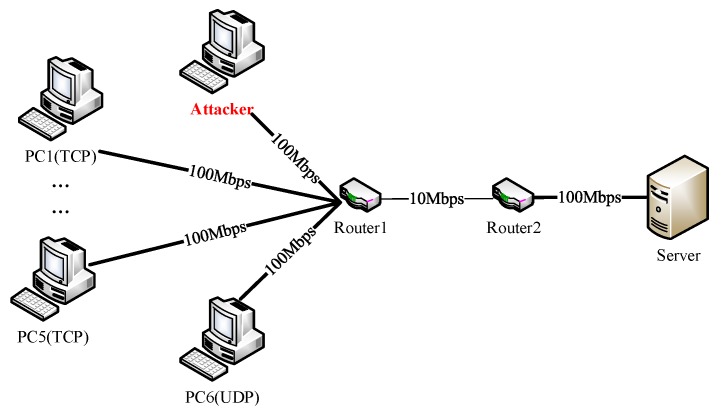
Topology of testbed.

For verifying the detection performance of this method for LDoS attacks in the real network environment, we established a network platform as Figure 13 presented. The testbed consisted of six legal users, one LDoS attacker, two routers and one server. There were six legal flows in the network, which included five TCP flows and one UDP flow. The bottleneck link bandwidth between router one and router two was 10 Mbps, and the remaining links bandwidth were 100 Mbps.

In this topology, six computers (PC1–PC6) used socket program to establish connections with the server. PC1–PC5 applied TCP protocol and PC6 adopted UDP protocol. All six computers sent packets to the server continuously. LDoS attacker reduced the quality of network service by sending high speed pulse stream periodically. We set the attack period to 1 s, and adjusted the attack intensity by changing the attack duration and rate. The attack rate was controlled by changing the number of threads. The larger the number of threads, the stronger the attack rate.

#### 5.2.2. Performance of LDoS Attacks Detection

We also conducted multiple sets of experiments on the testbed to evaluate the performance of our algorithm. The specific experimental parameters were set as shown in Table 2. Sampling time and duration of detection window were set as the same as NS2. Then the time-frequency joint distribution of network traffic in a detection window was obtained by STFT. The feature matrix was calculated. We used the matrix extracted from the training data to construct the isolation forest. The isolation forest was used to calculate the anomaly score of the four groups of test data.

**Figure 14:**
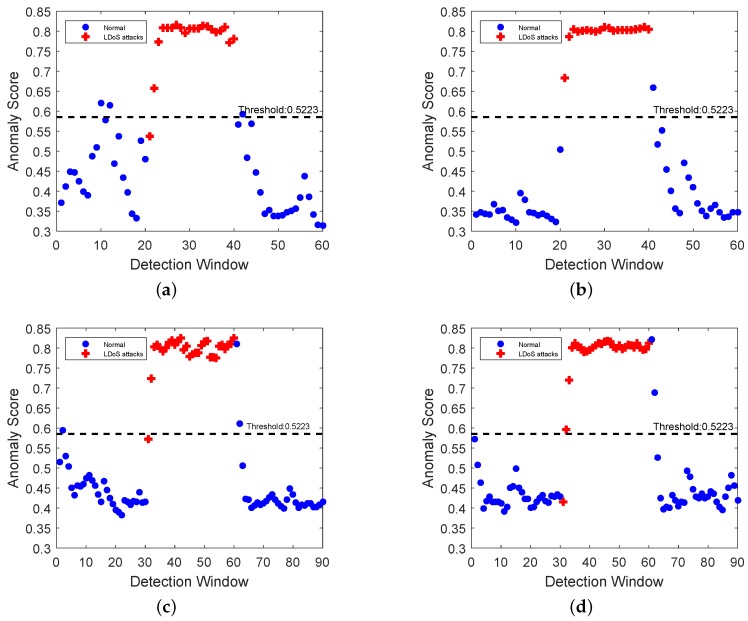
Detection results of four groups in testbed: (**a**–**d**) are corresponding to the detection results of G1, G2, G3, and G4 respectively.

**Figure 15:**
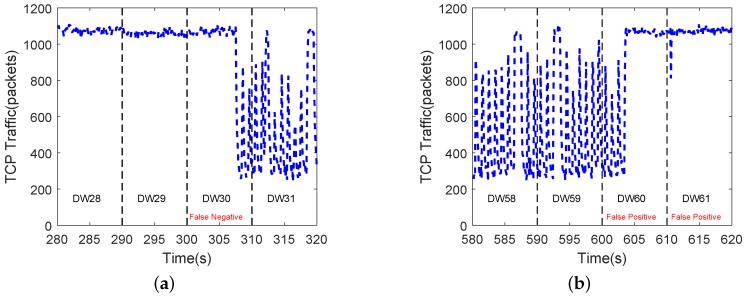
TCP traffic in G4 of testbed: (**a**) depicts TCP traffic at the start of LDoS attacks, and (**b**) shows TCP traffic at the end of LDoS attacks.

Among the four groups of test data, the method proposed in this paper could identify most of the attack data as presented in Figure 14. The misjudgment mainly occurred at the moment when the attack just began or ended. We have analyzed network traffic of G4 in Figure 15. The network traffic was in the transition stage between LDoS attacks state and normal state so that it was wrongly judged.

### 5.3. Experiments on Public Datasets LBNL and WIDE2018

We performed experiments on the Measurement and Analysis on the WIDE Internet (WIDE2018) dataset and the Lawrence Berkeley National Laboratory (LBNL) dataset in this subsection. These datasets consisted of various speed of links from 2 Mbps to 10 Gbps. Neither of the above two datasets contained LDoS attacks, so we used only the false positive rate to evaluate the detection performance.

**Figure 16:**
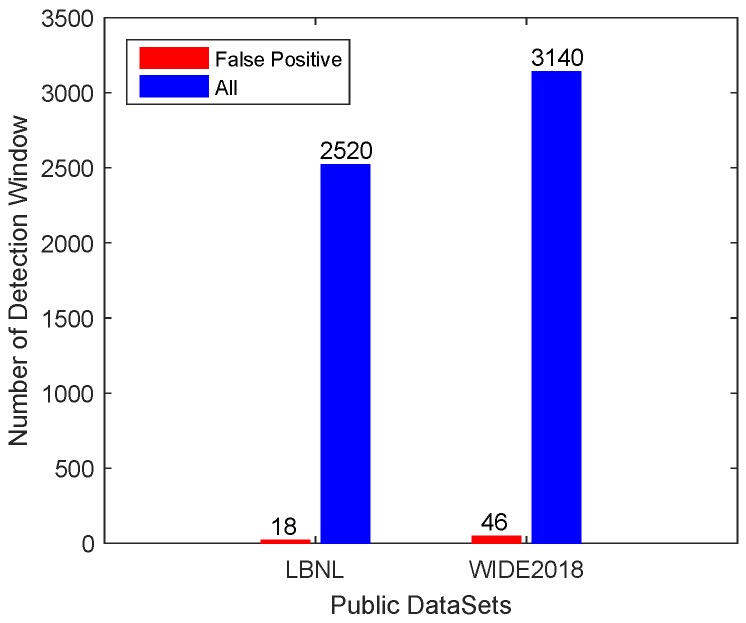
Detection results in public datasets.

From WIDE2018, we selected 16 days of data (from 20180101 to 20180216) and used 35 days of data (from 20180217 to 20180528) for training. In LBNL, 21 days of data were selected and the first 600 s of each day was used for training. We applied methods to classify the network traffic, and the classification results are shown in Figure 16. When classifying these real normal TCP traffic, our method generated 46 false alarms on WIDE2018, and the false positive rate on LBNL was only 0.71%.

### 5.4. Comparison with Other LDoS Methods

The detection method in this paper is compared with the existing algorithms for detecting LDoS attacks in recent years in terms of experimental platform, detection accuracy, false negative rate and false positive rate as shown in Table 3. It shows that our method could effectively detect LDoS attacks of different intensity on NS2 and testbed. On top of that, detection performance of LBNL and WIDE also proves that our method could overcome the influence of noise and had a low false alarm rate in the real complex network environment. Compared with other methods, the method we proposed had better detection performance in terms of higher detection accuracy and lower false positive rate.

## 6. Conclusions and Future Work

In this paper, we analyzed that the statistic attributes of TCP traffic in the time-frequency joint domain would be changed under LDoS attacks. Based on that, we developed MAF-ADM for LDoS attacks. On the one hand, the weighted fusion algorithm was applied to build the isolation forest according to the ability of the isolation trees to isolate samples containing LDoS attacks. On the other hand, we adopted the weighted moving average algorithm and the dynamic threshold algorithm to calculate anomaly score and threshold according to different network environments.

The method we proposed could detect 100% of LDoS attacks successfully on simulation platforms NS2, which does have good sensibility to identify LDoS attacks of different parameters. Results of experiments on testbed and the public datasets also demonstrate that this method does have better adaptability in the complex real network environment and immune to normal fluctuations of the network traffic.In conclusion, the proposed method can distinguish LDoS attacks and legitimate traffic effectively. It has better adaptability, higher accuracy and lower false positive rate.

In the future work, we will continue our research in two directions. First, we will put effort to study variations of LDoS attacks and how they work, such as the aggregated or synchronous low-rate distributed DoS attacks. Another promising direction we hope to achieve is the development of MAF-ADM to defend against variants of LDoS attacks and the deep integration of MAF-ADM with other network security appliances against LDoS attacks, such as intrusion detection in wireless sensor network, prevention appliance in cloud computing, and so forth.

## Figures and Tables

**Table 1:** Experimental parameters of NS2.

Group		Time (s)	Attack Parameters		
			R (Mbps)	P (s)	L (s)
Train	G0	2250	–	–	–
Test	G1	750	–	–	–
	G2	1210	[2,30]	1	0.1
	G3	1320	8	[0.4,8]	0.2
	G4	1430	8	1	[0.1,0.9]

**Table 2:** Experimental parameters of testbed.

Group		Sum Time (s)	Time of LDoS Attacks (s)	Attack Parameters		
				R (Thread Count)	P (s)	L (s)
Train	G0	800	–	–	–	–
Test	G1	600	200–400	500	1	0.2
	G2	600	200–400	750	1	0.2
	G3	900	300–600	1000	1	0.1
	G4	900	300–600	1000	1	0.1

**Table 3:** Detection performance compared with other methods.

Detection System	Simulated/Real Environment	Detection Performance (%)		
		Detection Accuracy	False Positive Rate	False Negative Rate
IIR [20]	Network Simulator 2(NS2)	81.36	7.45	18.64
Adaptive KPCA [39]	Network Simulator 2(NS2)	99.2	2	0.8
Kalman Filtering [31]	Testbed	89.6	12.6	10.4
FCE [40]	Testbed	90.02	4.3	9.98
Multifractal [28]	Network Simulator 2(NS2)	92	9	8
	Testbed	91	10	9
FPSE [23]	Network Simulator 3(NS3)	95.32	0.18	4.68
	Public Datasets (WIDE)	–	5.876	–
Two-step Cluster [30]	Network Simulator 2(NS2)	NA	NA	NA
	Public Datasets (WIDE2018)	–	5.56	–
	Public Datasets (LBNL)	–	2.46	–
Our method	Network Simulator 2(NS2)	100	0.13	0
	Testbed	97	4.5	3
	Public Datasets (WIDE2018)	–	1.46	–
	Public Datasets (LBNL)	–	0.71	–

NA = Not Available; – = Not Exist; 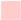 Testbed 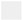 Public Datase.
